# An effector from the Huanglongbing-associated pathogen targets citrus proteases

**DOI:** 10.1038/s41467-018-04140-9

**Published:** 2018-04-30

**Authors:** Kelley Clark, Jessica Yvette Franco, Simon Schwizer, Zhiqian Pang, Eva Hawara, Thomas W. H. Liebrand, Deborah Pagliaccia, Liping Zeng, Fatta B. Gurung, Pengcheng Wang, Jinxia Shi, Yinsheng Wang, Veronica Ancona, Renier A. L. van der Hoorn, Nian Wang, Gitta Coaker, Wenbo Ma

**Affiliations:** 1 Department of Microbiology and Plant Pathology, University of California, Riverside, 92521 CA USA; 2 Department of Plant Pathology, University of California, Davis, 95616 CA USA; 3 Center for Plant Cell Biology, University of California, Riverside, 92521 CA USA; 4 Citrus Research and Education Center, University of Florida, Lake Alfred, 33850 FL USA; 5 Citrus Center, Texas A&M University, Weslaco, 78599 TX USA; 6 Department of Chemistry, University of California, Riverside, 92521 CA USA; 7 University of Oxford, Oxford, OX1 2JD UK; 8 Present Address: College of Life and Environmental Sciences, Shanghai Normal University, Shanghai, 200234 China

## Abstract

The citrus industry is facing an unprecedented challenge from Huanglongbing (HLB). All cultivars can be affected by the HLB-associated bacterium *‘Candidatus* Liberibacter asiaticus’ (*C*Las) and there is no known resistance. Insight into HLB pathogenesis is urgently needed in order to develop effective management strategies. Here, we use Sec-delivered effector 1 (SDE1), which is conserved in all *C*Las isolates, as a molecular probe to understand *C*Las virulence. We show that SDE1 directly interacts with citrus papain-like cysteine proteases (PLCPs) and inhibits protease activity. PLCPs are defense-inducible and exhibit increased protein accumulation in *C*Las-infected trees, suggesting a role in citrus defense responses. We analyzed PLCP activity in field samples, revealing specific members that increase in abundance but remain unchanged in activity during infection. *SDE1*-expressing transgenic citrus also exhibit reduced PLCP activity. These data demonstrate that SDE1 inhibits citrus PLCPs, which are immune-related proteases that enhance defense responses in plants.

## Abstract (web summary)

Greening disease threatens the productivity of citrus crops worldwide yet the pathosystem is poorly understood. Here, Clark et al. show that an effector cloned from the associated bacteria can suppress host plant papain-like cysteine proteases' activity, suggesting its probable role in pathogenesis.

## Introduction

Huanglongbing (HLB), or citrus greening disease, is currently considered the most destructive disease of citrus worldwide^1–5^. In the major citrus-growing areas including the US and Asia, the presumed causal agent of HLB is a Gram-negative bacterium, *‘Candidatus* Liberibacter asiaticus’ (*C*Las). *C*Las is transmitted to citrus by the Asian citrus psyllid (ACP) during sap feeding, where it then colonizes the phloem sieve elements, eventually leading to disease symptoms. Infected trees exhibit leaf mottling, deformed/discolored fruits, premature fruit drop, and premature mortality^2^. In the US, Florida has lost over $7 billion in total industry output due to HLB since it was first detected in 2005 till 2014^6,7^.

Secreted proteins of pathogens, called effectors, play an essential role in bacterial pathogenesis. Collectively, effectors aid infection by suppressing plant immunity and creating environments favorable for colonization and proliferation^8,9^. Many Gram-negative bacteria ‘inject’ effectors directly into host cells through the type III secretion system^10^. In contrast, insect-transmitted bacteria, such as *C*Las, often lack this specialized delivery machinery, but can utilize the general Sec secretion system to release effectors^11^. These Sec-delivered effectors (SDEs) carry an N-terminal secretion signal, allowing their export from pathogen cells into the extracellular space. The essential roles of SDEs in bacterial virulence are best illustrated by insect-transmitted, phloem-colonizing phytoplasmas, where expression of their individual SDEs in *Arabidopsis thaliana* leads to phenotypes that mimic disease symptoms^12,13^. Sequence analysis of the *C*Las genome revealed that it encodes all the components of the Sec secretion machinery^14^. In addition, 86 proteins were confirmed to possess a functional Sec-secretion signal, indicating that they could potentially be released by *C*Las into the phloem during infection^15^. A few of these SDEs exhibited higher expression levels in citrus relative to their levels of expression in ACP^14,15^, indicating that they may contribute to *C*Las colonization and/or disease progression in citrus. However, our knowledge on the cellular function of *C*Las SDEs in plant or insect hosts is lacking.

Here, we characterize the *C*Las effector SDE1 (CLIBASIA_05315) and identify its targets in citrus. SDE1 is conserved across *C*Las isolates with a typical Sec-dependent secretion signal^15–17^. The expression of *SDE1* is ~10-fold higher in citrus than in ACP^16^, indicating a role in *C*Las colonization of plant hosts. *SDE1* is also highly expressed in asymptomatic tissues, suggesting a potential virulence function during early infection stages. We show that SDE1 interacts with multiple members of papain-like cysteine proteases (PLCPs), which are known to regulate defense in *Arabidopsis* and solanaceous crops against bacterial, fungal, and oomycete pathogens^18,19^. The abundance of PLCPs is increased in citrus infected with *C*Las, likely as a defense response. Interestingly, SDE1 can directly inhibit PLCP activity in vitro and in citrus. Using a surrogate system, we further show that SDE1 is able to promote bacterial infection in *Arabidopsis*. Taken together, this research advances our understanding of HLB pathogenesis by identifying citrus targets of a conserved *C*Las effector, which could be exploited for HLB management.

## Results

### SDE1 associates with citrus papain-like cysteine proteases

**Fig. 1:**
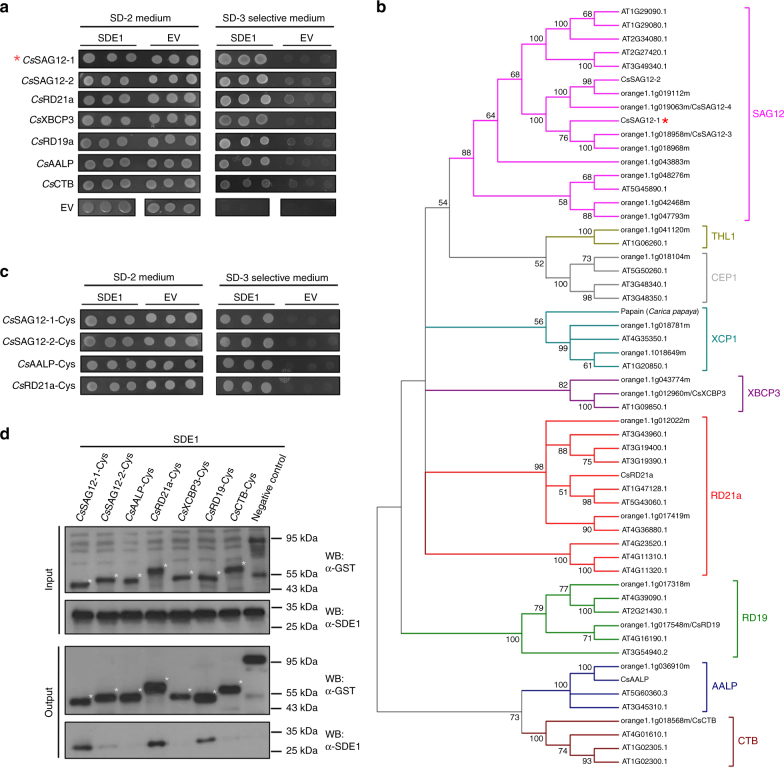
SDE1 interacts with citrus papain-like cysteine proteases. **a** Yeast-two-hybrid (Y2H) assays using the *C*Las effector SDE1 as the bait and full-length citrus papain-like cysteine proteases (*Cs*PLCPs), representing different subfamilies as the prey. SDE1 was cloned into the vector pGBKT7 and individual *Cs*PLCPs were cloned into the vector pGADT7. Growth of yeast cells on SD-3 selective media represents protein–protein interaction, growth of the same cells on SD-2 media confirms yeast transformation. Yeast transformed with the empty vectors served as negative controls. The initial PLCP found from Y2H screening (*Cs*SAG12-1, XM_006495158) is indicated with an asterisk (*). The gene IDs of the other interacting PLCPs are *Cs*SAG12-2 (XM_006470229), *Cs*XCBP3 (orange1.1g012960), *Cs*RD21a (XM_006473212), *Cs*RD19 (orange1.1g017548), *Cs*AALP (XM_006474664), and *Cs*CTB (orange1.1g018568). **b** Phylogeny and subfamily classification of canonical PLCPs in the *C*. *sinensis* (sweet orange) genome. The phylogenetic tree was made with MEGA6.06 (100 bootstrap replicates, Maximum-Likelihood method, Jones–Taylor–Thornton model), using the *Arabidopsis thaliana* PLCP subfamily classification^22^. The asterisk (*) indicates the initially found *Cs*SAG12-1. **c** Y2H assay examining the interaction of SDE1 with the cysteine protease domain of *Cs*PLCPs. **d** In vitro pull-down assay using the GST-tagged cysteine protease domain of *Cs*PLCPs to immunoprecipitate SDE1 protein. Input and immunoprecipitated proteins (output) were visualized by western blotting using anti-GST and anti-SDE1 antibodies. Asterisks (*) indicate the protein bands that correspond to individual *Cs*PLCPs. GST-tagged *Arabidopsis* double-stranded DNA-binding protein 4 (*At*DRB4) was used as a negative control

SDE1 is unique to *C*Las with no homologs in other organisms^16^. It is found in all sequenced *C*Las isolates from various geographic regions and its expression was detected from *C*Las-infected citrus varieties including limes, sweet oranges, and grapefruits^15,16^. To understand the potential virulence function of SDE1 in citrus, we performed sequencing-based yeast-two-hybrid (Y2H) screening using a *Citrus sinensis* cDNA library to identify candidate SDE1-interacting proteins (Supplementary Table 1). A selection of these candidates was further examined using a pair-wise Y2H assay. Of the six evaluated candidates, the *C*. *sinensis* protein annotated as ‘xylem cysteine protease 1’ (NCBI accession XM_006495158, previously GI# 568885285) was confirmed by Y2H as interacting with SDE1 (Fig. 1a).

Xylem cysteine protease 1 is a member of the papain-like cysteine protease (PLCP) family. PLCPs share a conserved protease domain including a catalytic triad consisting of cysteine, histidine, and asparagine^19^ (Supplementary Fig. 1a). The canonical PLCPs have a pro-domain that must be autocatalytically processed for activity. The pre-proteases often contain an N-terminal signal peptide to ensure their entrance into the endomembrane system and subsequent function in the apoplast, vacuole, or lysosomes (Supplementary Fig. 1a). Previous reports have shown that PLCPs contribute to plant defense during bacterial, oomycete, and fungal infection^19–21^. Search of the *C*. *sinensis* genome revealed 21 canonical PLCPs that can be classified into nine subfamilies based on their homology to the previously categorized *Arabidopsis* PLCPs^22^ (Fig. 1b). Based on our phylogenic analysis, XM_006495158 belongs to the SAG12 subfamily and is hereafter referred to as *Cs*SAG12-1. Structural modeling using CysEP, a castor oil (*Ricinus communis*) PLCP involved in programmed cell death (PCD)^23^, indicates that *Cs*SAG12-1 adopts a similar fold in the protease domain, further supporting it as a PLCP (Supplementary Fig. 1b).

Since PLCPs share a conserved catalytic domain, we examined whether SDE1 could also associate with PLCPs from other subfamilies. Representatives from five additional PLCP subfamilies, *Cs*XCBP3 (orange1.1g012960), *Cs*RD21a (XM_006473212), *Cs*RD19 (orange1.1g017548), *Cs*AALP (XM_006474664), and *Cs*CTB (orange1.1g018568), were tested. Remarkably, all of them were able to interact with SDE1 in yeast (Fig. 1a). Furthermore, a second member of the SAG12 subfamily, *Cs*SAG12-2 (XM_006470229), also interacted with SDE1 (Fig. 1a). The observation that SDE1 interacts with members from multiple PLCP subfamilies suggests that it may associate with the conserved protease domain. Indeed, the protease domains of *Cs*SAG12-1, *Cs*SAG12-2, *Cs*RD21a, and *Cs*AALP are sufficient to mediate interaction with SDE1 in yeast (Fig. 1c). In addition, SDE1 interacted with the protease domains of three other members from the SAG12 subfamily, i.e. *Cs*SAG12-3 (orange1.1g018958), *Cs*SAG12-4 (orange1.1g019063), and *Cc*SAG12-1 (Ciclev10005334, a PLCP from *C*. *clementina*) in yeast (Supplementary Fig. 2).

In order to determine whether SDE1 can directly interact with citrus PLCPs, we conducted in vitro pull-down assays using recombinant proteins expressed and purified from *Escherichia coli*. The protease domains of the PLCPs were tagged with GST at the N-terminus and the recombinant proteins were incubated with HIS-tagged SDE1 in excess. The protein complexes were immunoprecipitated using glutathione beads and enrichment of SDE1 was detected by western blotting. Our results show that SDE1 co-precipitated with the protease domains of *Cs*SAG12-1, *Cs*SAG12-2, *Cs*RD19, and *Cs*RD21a (Fig. 1d). Although *Cs*AALP, *Cs*XCBP3, and *Cs*CTB were able to interact with SDE1 in yeast, these interactions were not detected in the in vitro pull-down assay. This could be, at least in part, due to the poor solubility of the recombinant GST-PLCP proteins when produced in *E*. *coli*. The cysteine residues within the protease domains have the potential to form disulfide bonds^19,22^, which may have resulted in incorrect folding and/or low solubility of these normally secreted PLCPs when expressed in the cytoplasm. Another possibility is that the pull-down assay is more stringent (and thus, less sensitive) in monitoring particular SDE1-PLCP interactions than Y2H. Nonetheless, these experiments strongly suggest that SDE1 can interact with multiple PLCPs belonging to different subfamilies through the conserved cysteine protease domain.

### SDE1 inhibits PLCP activity

Knowing SDE1 interacts with PLCPs through the protease domain, we next examined whether it could inhibit their proteolytic activity. Several assays were used to measure the proteolytic activities of PLCPs in the presence of SDE1. In all these assays, the chemical inhibitor E-64, which forms a covalent bond with the catalytic cysteine of the PLCP protease domain, was used as a positive control^24^.

**Fig. 2:**
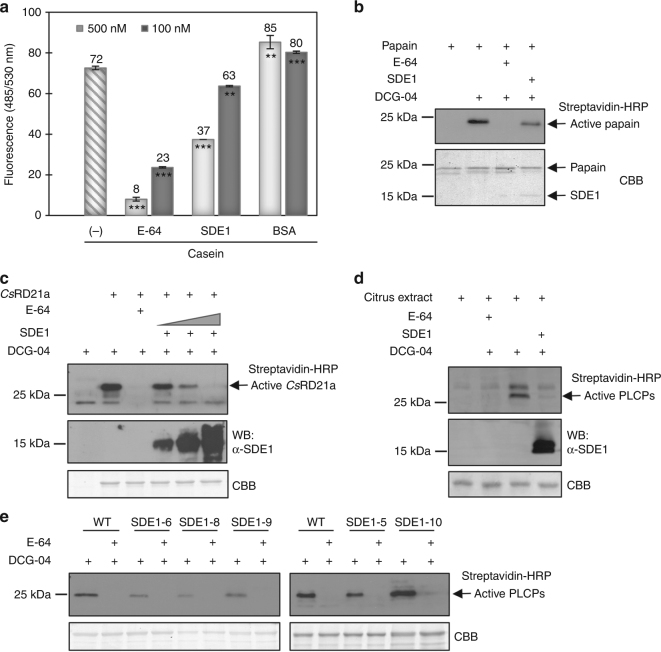
SDE1 inhibits PLCP activity in vitro and in plant cells. **a** Proteolytic activity of papain measured by digestion of a fluorescent casein substrate in the presence of E-64, purified SDE1 protein, or BSA (as a negative control). Fluorescence was measured at 485/530 nm excitation/emission. Mean ± standard deviation (*n* = 3) is shown. Asterisks (*) indicate statistically significant differences based on the two-tailed Student’s *t*-test. *p* < 0.01 = **, *p* < 0.001 = ***. **b** Inhibitory effect of SDE1 on the protease activity of papain examined by activity-based protein profiling (ABPP). Active papain was labeled by DCG-04 in the presence of 10 μM E-64 or 1.6 μM purified SDE1 protein and detected using streptavidin conjugated with horseradish peroxidase (HRP). **c** SDE1 inhibits the activity of *Cs*RD21a. *Cs*RD21a-Flag (with its N-terminal secretion signal) was expressed in *N*. *benthamiana*. Active protease in the apoplastic fluid was labeled via ABPP. ImageJ analysis of the signal intensity revealed approximately 9%, 62%, and 96% reduction of *Cs*RD21a activity in the presence of 0.8, 1.6, or 3.2 μM purified SDE1 protein, respectively. **d** SDE1 inhibits PLCP activity in citrus. Total protein extracts from Navel orange (*C*. *sinensis*) leaves were labeled via ABPP in the presence of 120 nM purified SDE1 protein. Active proteases were enriched using streptavidin beads and detected using streptavidin-HRP conjugates. **e** Transgenic grapefruit (Duncan) seedlings expressing *SDE1* exhibit reduced protease activity. Five individual lines were analyzed by ABPP. SDE1-10 does not have significant SDE1 protein accumulation and served as a negative control

First, we examined the inhibitory effect of SDE1 on the proteolytic activity of papain, a PLPC from papaya^22^. Fluorescein-labeled casein was used as a substrate which, upon cleavage by papain, releases a fluorescent signal that can be quantified using a fluorometer. Our results show that SDE1 inhibited substrate cleavage by papain in a dose-dependent manner (Fig. 2a). Using 100 and 500 nM purified SDE1 protein, the proteolytic activity of papain was decreased by 12% and 49%, respectively, when compared to papain alone. This inhibitory effect is significant, although weaker compared to that of E-64, which reduced protease activity at the same concentrations by about 68% and 85%. As a negative control, addition of BSA or another *C*Las effector, termed SDE2, which does not interact with PLCPs, did not reduce the protease activity of papain (Fig. 2a, Supplementary Fig. 3).

Next, we examined whether SDE1 binds near the catalytic site of PLCPs, if so, its interaction with PLCPs should be blocked by pre-incubation with E-64. We conducted in vitro pull-down assays with or without E-64 using the protease domains of two citrus PLCPs, *Cs*SAG12-1 and *Cs*RD21a, that can be pulled down by SDE1 in the absence of E-64 (Fig. 1d). We also included a third PLCP, Resistance to *Cladosporium fulvum* 3 (RCR3), which is a member of the tomato SAG12 subfamily and is known to be inhibited by the Avr2 effector from the fungal pathogen *C*. *fulvum*^25^. The protease domains of these PLCPs were expressed in *E*. *coli* and enriched using GST affinity resins. PLCP-bound resins were pre-incubated with 200 μM E-64 and the enrichment of SDE1 with the resins was examined by western blotting. Co-precipitation of SDE1 with all three PLCPs was reduced in the presence of E-64, suggesting that SDE1 binds near the catalytic cysteine bound by E-64, resulting in a steric hindrance around the active site (Supplementary Fig. 4). Since SDE1–PLCP interactions were not completely abolished by the addition of E-64, it is likely that SDE1 does not directly bind to the catalytic cysteine residue. Rather, SDE1 might block the catalytic cleft to prevent access to substrates, thus inhibiting proteolytic activity. Alternatively, the binding of E-64 to the catalytic cysteine could result in conformational changes of the protease, and therefore, partially interfere with SDE1’s interaction with the PLCPs.

Finally, we directly measured the protease activity of SDE1-interacting PLCPs using activity-based protein profiling (ABPP) where DCG-04, a biotinylated derivative of E-64, is used as a probe^24^. Since E-64 only binds to the active form of cysteine proteases, western blots using streptavidin conjugated with horseradish peroxidase (HRP) can detect DCG-04-labelled PLCPs via biotin, and the signal intensity reflects their activity level. First, we examined ABPP of papain in the presence of SDE1 or E-64. Our results showed that pre-incubation with SDE1 at 1.6 μM was able to reduce DGC-04 labeling by about 53%, demonstrating that SDE1 suppresses the protease activity of papain in vitro (Fig. 2b). Pre-incubation of papain with E-64 (10 μM) completely abolished the DCG-04 labeling, which is consistent with the results from the in vitro protease activity assay using the fluorescein-labeled substrate.

We also conducted ABPP in a semi-in vitro assay using recombinant SDE1 protein purified from *E*. *coli* and PLCPs expressed in plant tissues. To this end, full-length *CsRD21a* was transiently expressed in *Nicotiana benthamiana* leaves. Using the native N-terminal secretion signal, *Cs*RD21a was secreted into the apoplast as shown by Coomassie brilliant blue (CBB) stain comparing control apoplastic fluids from wild-type *N*. *benthamiana* to those transiently expressing *Cs*RD21a (Fig. 2c). *Cs*RD21a could be labeled by DCG-04, suggesting that it is an active enzyme. A reduction of *Cs*RD21a activity was observed with the addition of SDE1 in a dose-dependent manner using 0.8, 1.6, or 3.2 μM purified proteins (Fig. 2c). We then determined whether SDE1 could inhibit other PLCPs in citrus. Total proteins were extracted from leaves of Navel oranges (*C*. *sinensis*). We induced PLCP accumulation by spraying the leaves with 2 mM of the defense hormone salicylic acid (SA)^26^, followed by total protein extraction and incubation with purified SDE1 protein. In this experiment, we further purified and concentrated the labeled PLCPs using streptavidin beads. Immunoblots using streptavidin-HRP showed that PLCP activity was greatly decreased after incubation with 120 nM SDE1, and completely inhibited with 25 μM E-64 (Fig. 2d). Together, these results demonstrate that SDE1 suppresses the protease activity of *Cs*RD21a and possibly other citrus PLCPs natively in the plant cells.

To further demonstrate that PLCPs are the in vivo targets of SDE1 in citrus, we generated transgenic seedlings of Duncan grapefruit expressing *SDE1* (without the N-terminal 1-24 aa that corresponds to a secretion signal peptide) under the cauliflower mosaic virus *35S* promoter. Total protein extracts from leaf tissues of 1-year-old seedlings were labeled with DCG-04 and the levels of active PLCPs were examined by western blotting using streptavidin-HRP. Our results show reduced PLCP activities in four independent *SDE1*-expressing lines (SDE1-5, SDE1-6, SDE1-8, and SDE1-9), relative to an untransformed control (Fig. 2e). We confirmed that these lines were indeed producing SDE1 proteins using western blotting (Supplementary Fig. 5). In addition, the transgenic line SDE1-10 exhibited little to no SDE1 protein accumulation (Supplementary Fig. 5), which correlated with a lack of reduction in protease activity in this line (Fig. 2e). Taken together, these data strongly suggest that SDE1 can inhibit the protease activity of PLCPs in citrus.

### Citrus PLCPs accumulate during SA treatment and infection

**Fig. 3:**
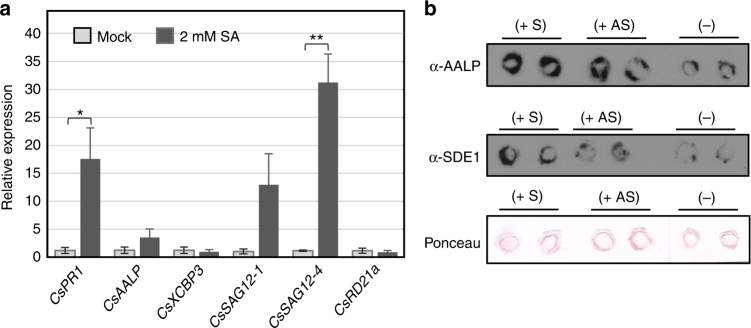
*Cs*PLCPs accumulate during SA treatment and infection. **a** Abundance of PLCP genes was determined by quantitative RT-PCR after SA treatment. One-year-old Navel oranges (*C*. *sinensis*) were sprayed with 2 mM SA or water. Leaf samples were collected after 48 h for RNA extraction and PCR analyses. Cytochrome oxidase subunit 1 (*COX*, KF933043.1) was used as the internal standard. Graph shows mean ± standard error of three replicates. Asterisks (*) indicate statistically significant differences based on the two-tailed Student’s *t*-test. *p* < 0.05 = *, *p* < 0.01 = **. **b** Protein abundance of PLCPs was determined in healthy (−) or *C*Las-infected (+) citrus branches using an anti-AALP antibody. Freshly cut stems were stamped onto nitrocellulose membranes and PLCPs and SDE1 were detected using western blotting. The titer of *C*Las in each sample was evaluated by quantitative PCR with observed Ct values of 27.97 for symptomatic tissue (+S), and not detected for asymptomatic tissue from the same infected tree (+AS) or tissue from an uninfected tree (−). Ponceau-stained membrane was shown as a control

In order to determine whether PLCPs are involved in defense-related responses in citrus, we looked at PLCP expression changes in both defense-induced and *C*Las-infected citrus. To activate defense signaling, leaves of Valencia oranges (*C*. *sinensis*) were sprayed with 2 mM salicylic acid (SA)^26^. The transcript abundance of five *Cs*PLCP genes was then determined by quantitative RT-PCR (qRT-PCR). Upon SA treatment, we detected an increase in the expression of *Pathogenesis-related gene 1* (*CsPR1*)^27^, which is a commonly used marker for the SA response. Although the magnitude of induction varied across trees, we consistently found a PLCP gene belonging to the SAG12 subfamily (*CsSAG12-4*) to be significantly up-regulated upon SA treatment (Fig. 3a). *CsSAG12-1* and *CsAALP* also showed a trend of increased expression in response to SA treatment, although the induction was not statistically significant. In addition, citrus PLCP genes have been shown to be transcriptionally induced in response to *C*Las infection^28,29^. Analysis of publicly available transcriptome data^28,29^ found genes encoding *Cs*PLCPs of several subfamilies including, but not limited to, SAG12, RD21a, and AALP to be upregulated during *C*Las infection (Supplementary Table 2). These results indicate that citrus PLCPs may act as defense proteases in *C*Las-infected trees.

Since *C*Las is a phloem-colonizing bacterium, we next assessed whether SDE1 and PLCPs could both be detected in the phloem sap of infected citrus trees. For this purpose, we performed direct tissue imprints using anti-SDE1^16^ or anti-AALP^30^ antibodies, respectively. We monitored AALP as a representative of PLCPs in this experiment due to the availability of the antibody, although induction of *CsAALP* by SA treatment was not as robust as induction of the *SAG12* subfamily members (Fig. 3a). The specificity of the anti-AALP antibody was verified using DCG-04 labeling followed by western blotting (Supplementary Fig. 6). Young stems from *C*Las-infected and healthy (i.e. *C*Las-free) trees of Rio Red grapefruit (*Citrus paradisi*) were freshly cut and imprinted onto nitrocellulose membranes, which were then incubated with either anti-SDE1 or anti-AALP. For the *C*Las-infected trees, we examined both symptomatic and asymptomatic tissues, which presumably represent late and early infection stages, respectively, as suggested by the bacterial titers. Our results show that while SDE1 was only present in the infected tissues, AALPs were detected from both healthy and infected tissues (Fig. 3b). However, the signals representing AALPs were stronger in the infected stems, both symptomatic and asymptomatic, compared to those from the healthy stems. This is consistent with the increased abundance of PLCP genes revealed by qRT-PCR of SA-treated citrus (Fig. 3a) and the analysis of previous transcriptome data (Supplementary Table 2). Furthermore, similar to SDE1, the AALP signals were mainly detected from the bark layers, which is enriched with phloem cells.

### Uncoupling PLCP abundance and activity during *C*Las infection

**Fig. 4:**
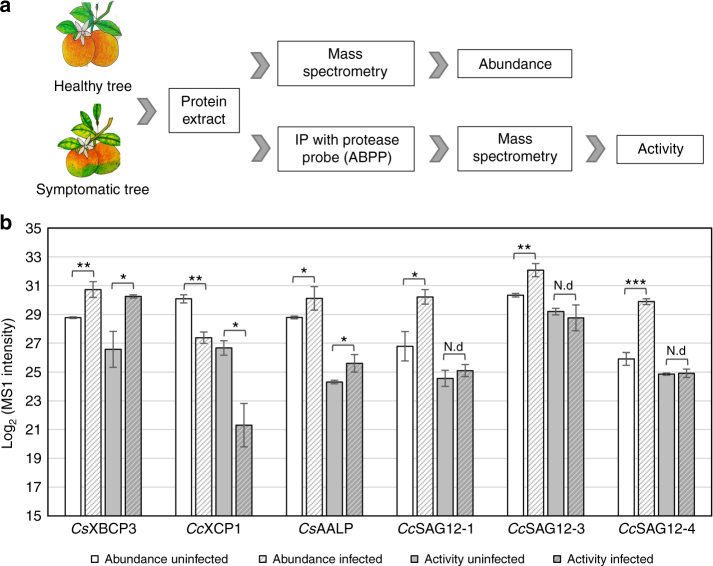
*Cs*SAG12s increase in abundance but not activity in infected citrus. **a** Diagram illustrating the experimental approach for detecting abundance and activity of *Cs*PLCPs in healthy and infected Navel oranges (*C*. *sinensis*) from a Texas grove using mass spectrometry. **b** Abundance and protease activity of six PLCPs belonging to four subfamilies in citrus leaf samples. Leaf tissue was ground in Tris buffer and divided into two to assess abundance and activity. PLCP abundance was tested using an in-solution digest coupled with mass spectrometry. For activity, the addition of DCG-04 permits the labeling of active PLCPs. Active PLCPs were captured and identified by streptavidin IP coupled with mass spectrometry. Mean ± standard error of three replicates is shown. Asterisks (*) indicate statistically significant differences based on the two-tailed Student’s *t*-test. N.d = no difference, *p* < 0.05 = *, *p* < 0.01 = **, *p* < 0.001 = ***. The gene IDs are *Cs*XBCP3 (orange1.1g012960m), *Cc*XCP1 (Ciclev10001665m), *Cs*AALP (orange1.1g036910m), *Cc*SAG12-1 (Ciclev10005334m), *Cs*SAG12-3 (orange1.1g018958m), and *Cs*SAG12-4 (orange1.1g019063m). Jessica Franco created the citrus images in panel **a** by hand for use in this paper

During pathogen recognition, PLCP abundance is usually increased alongside their activity^31^. Previous studies have demonstrated that various pathogens can selectively inhibit PLCPs in their specific plant hosts to facilitate disease progression^19^. To determine whether this occurs during *C*Las infection, we performed comparative proteomics using tissues from mature Navel orange (*C*. *sinensis*) trees grown in a Texas grove. Leaves from *C*Las-infected (symptomatic) trees were collected. As a control, uninfected leaf samples were collected from trees held in a screenhouse that was consistently tested for *C*Las by qRT-PCR. PLCP abundance in total protein extracts was determined by mass spectrometry (MS), while active protease levels were also analyzed in the same samples using ABPP coupled with MS quantification (Fig. 4a). We were able to detect multiple PLCP subfamilies by MS (Fig. 4b). Among them, members of the AALP and XBCP3 subfamilies significantly increased in abundance as well as activity in infected trees compared to uninfected controls. A member of the XCP1 subfamily exhibited decreased abundance as well as activity in infected trees. Interestingly, the abundance and activity did not correlate for the three SAG12 subfamily members in this analysis. The abundance of *Cc*SAG12-1, *Cs*SAG12-3, and *Cs*SAG12-4 significantly increased in infected trees, whereas their activity remained unchanged (Fig. 4b). This result indicates that these SAG12 subfamily members are potentially involved in citrus defense responses and that their activities might be inhibited by *C*Las. While it is tempting to speculate that SDE1 contributes solely to the inhibition of these PLCPs, the observed effect could be due to the concerted action of several effector proteins and/or other virulence factors of *C*Las, in addition to SDE1.

### SDE1 promotes bacterial infection

**Fig. 5:**
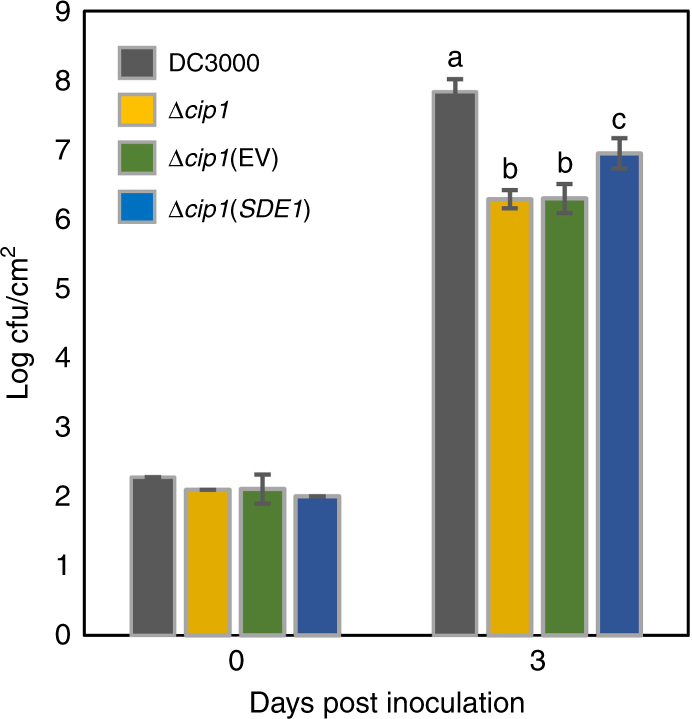
SDE1 promotes *Pseudomonas syringae* infection of *Arabidopsis*. Mature leaves of 5-week-old plants of *Arabidopsis thaliana* ecotype Col-0 were syringe-infiltrated with cell suspensions of *P*. *syringae* pv. *tomato* (*Pto*) strains including DC3000 (wild type), Δ*cip1*, Δ*cip1*(empty vector, EV), and Δ*cip1*(*SDE1*). Bacterial titers were determined as colony forming units (cfu/cm^2^) at the time of infiltration (Day 0) and 3 days post infiltration (Day 3). Graph shows mean ± standard deviation of data from three independent experiments. Different letters (a, b, and c) indicate statistically significant differences (*p* < 0.05) based on a one-way ANOVA followed by Tukey’s HSD post hoc test

Despite substantial research efforts, *C*Las has not been successfully cultivated. In order to explore the potential contribution of SDE1 to bacterial virulence, we employed another Gram-negative bacterial pathogen, *Pseudomonas syringae*, as a surrogate. In particular, *P*. *syringae* pv. *tomato* strain DC3000 (*Pto*DC3000) was previously reported to produce a Sec-secreted protein called Cip1, which can inhibit the protease activity of tomato C14, a member of the RD21a subfamily of PLCPs^32^. A *cip1* knockout mutant of *Pto*DC3000 exhibited reduced virulence, indicating that Cip1 contributes to bacterial infection, likely through its inhibitory effect on PLCP activities^32^. We examined whether SDE1 could complement the Cip1 virulence activity that was lost in the knockout mutant of *Pto*DC3000. *SDE1* (full-length, containing its native Sec-secretion signal) was expressed in *Pto*DC3000Δ*cip1* under the promoter of *hopZ1a*, a type III-secreted effector that is activated during infection^33^. We were able to detect SDE1 protein in the supernatant of induced bacterial cell cultures, confirming that it was secreted by *P*. *syringae* (Supplementary Fig. 7). *Pto*DC3000, *Pto*DC3000Δ*cip1*, and two *Pto*DC3000Δ*cip1* strains either expressing *SDE1* or transformed with the empty vector (EV) were used to inoculate mature leaves of *Arabidopsis thaliana* ecotype Col-0 and the bacterial populations were determined 3 days post inoculation. The results show that while *Pto*DC3000Δ*cip1* and the EV control exhibited strong reductions in virulence compared to wild-type *Pto*DC3000, expression of *SDE1* partially, but significantly, complemented this virulence deficiency (Fig. 5). Collectively, these results suggest that SDE1 promotes bacterial infection, likely by inhibiting PLCP activity in plant hosts.

### SDE1 does not inhibit RCR3 activity in solanaceous plants

**Fig. 6:**
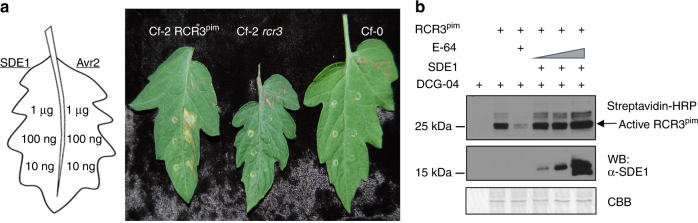
SDE1 does not inhibit Solanaceous RCR3 activity. **a** SDE1 did not elicit cell death in tomato (Moneymaker, *Solanum lycopersicum*) leaves expressing the immune receptor Cf-2. Purified recombinant proteins FLAG-Avr2-6XHIS or FLAG-6XHIS-SDE1 were infiltrated in tomato leaves in three different concentrations: 1 μg, 100 ng, and 10 ng. Avr2 inhibits the protease activity of RCR3^pim^ (RCR3 from *Solanum pimpinellifolium*, which is required for Avr2-triggered cell death in tomato plants expressing Cf-2 produced by *S*. *pimpinellifolium* (left). Cf-0 (right) and Cf-2 *rcr3* (middle, containing a *rcr3* mutant with a premature stop codon) tomatoes were used as negative controls. Picture was taken after 7 dpi. **b** SDE1 does not inhibit the activity of RCR3^pim^. Full length of RCR3^pim^-HIS was transiently expressed in *N*. *benthamiana* and secreted into the apoplast. Apoplastic fluid was extracted and the active protease was labeled via ABPP in the presence of 0.8, 1.6 or 3.2 μM of purified SDE1 protein. Coomassie brilliant blue (CBB) served as a loading control

In tomato, inhibition of RCR3 activity by the *C*. *fulvum* effector Avr2 activates Cf-2-mediated immune responses, including programmed cell death, conferring resistance to the fungal pathogen^25^. SDE1 interacts with RCR3 in vitro (Supplementary Fig. 4). We therefore tested whether SDE1 can likewise trigger Cf-2-mediated cell death in tomato. To this end, we infiltrated near-isogenic lines of tomato cultivar Moneymaker^25^ containing either Cf-2 and RCR3 (*Cf-2 RCR3*^*pim*^), Cf-2 only (*Cf-2 rcr3-3*), or lacking Cf-2 (Cf-0) with purified SDE1 protein (Fig. 6a). As a control, we infiltrated the same leaves with purified Avr2 protein. As expected, Avr2 triggered cell death in a Cf-2- and RCR3-dependent manner 7 days post infiltration; in contrast, no cell death was observed from SDE1-infiltrated areas even at high protein concentrations (Fig. 6a).

Next, we tested whether this lack of cell death was due to SDE1 not being able to inhibit RCR3. We performed ABPP using RCR3 from tomato (RCR3^pim^) and from the wild potato species *Solanum demissum* (RCR3^dms3^)^34^. Unlike with *Cs*RD21a, SDE1 was unable to inhibit the activity of either of the RCR3 proteins (Figs. 2c and 6b, Supplementary Fig. 8). This result indicates that the lack of Cf-2-mediated cell death in response to SDE1 is likely due to the inability of the *C*Las effector to inhibit the protease activity of RCR3 from these non-host plants and illustrates the host-specific function of SDE1.

## Discussion

The devastating impact of HLB on the citrus industry warrants immediate yet sustainable solutions, which we are only beginning to unveil. Advances in understanding of the molecular interactions between *C*Las and citrus will provide the fundamental knowledge needed to develop robust HLB management techniques. In this study, we used the effector SDE1 as a molecular probe to reveal PLCPs as virulence targets of *C*Las in citrus, thereby providing one of the first mechanistic insights into HLB pathogenesis.

PLCPs have been reported to regulate plant immunity and contribute to defense against a broad range of pathogens including bacteria, fungi, and oomycetes^20,21,25,32^. For example, the SAG12 subfamily members, RCR3 and PIP1, in tomato contribute to defense against the oomycete pathogen *Phytophthora infestans*^25,35^. Knocking out or silencing specific *PLCP* genes in *Arabidopsis*, tomato, and *N*. *benthamiana* resulted in increased susceptibly to various pathogens^19,36^. The mechanisms underlying PLCP-mediated defense could work on multiple levels. They may directly hydrolyze pathogen components—for example, growth inhibition by papain against the papaya pathogen *Phytophthora palmivora* was recently reported^37^. However, we did not observe an inhibitory effect of papain on bacterial growth in artificial media (Supplementary Fig. 9), suggesting that direct antimicrobial activity by PLCPs is highly specific. It is possible that PLCPs contribute to the citrus response to *C*Las by regulating defense signaling. For example, it was proposed that PLCPs could cleave microbial or host peptides to elicit defense responses^19^.

Bacterial, fungal, and oomycete pathogens as well as nematodes have all evolved effector proteins to suppress PLCP activities in order to promote infection^20,21,25,32,34,38,39^. Cip1 produced by the bacterial pathogen *P*. *syringae* is required for full virulence^32^. Similarly, the *C*. *fulvum* effector Avr2 and the *Ustilago maydis* effector Pit2 also play important roles during fungal infection of their respective plant hosts^40,41^. SDE1 was able to partially substitute for Cip1 function during infection, indicating that it might similarly promote *C*Las infection in citrus. Although PLCPs are a major hub of effector targets, none of these effectors share sequence similarities, suggesting that they have evolved independently (through convergent evolution) to interfere with the activities of this important group of defense regulators.

Phloem sieve tube elements are metabolically inactive and are supported by adjacent companion cells derived from the same mother cell^42^. PLCPs have been identified in phloem proteomic analyses of other plants, indicating that they could be directly secreted into sieve elements from adjacent companion cells^43,44^. We detected increased accumulation of AALP, XBCP3, and SAG12 subfamily members in *C*Las-infected citrus trees. We found that SDE1 associates with multiple *Cs*PLCPs in various subfamilies and there is a discordance between abundance and activity of three SAG12 members during *C*Las infection. SDE1 is potentially secreted into the phloem by *C*Las during infection, where it might act to suppress PLCP activity. SDE1 might also be able to move through the sieve elements and translocate into the companion cells via plasmodesmata to inhibit these important defense proteins (Supplementary Fig. 10). Further experiments are needed to investigate the mechanisms by which PLCPs contribute to citrus defense signaling and enhance immune responses to *C*Las.

The findings described in this work lay the foundation for the development of HLB-resistant germplasm through genetic manipulation. Our results showing that SDE1 does not inhibit RCR3 activity and thus fails to trigger Cf-2-dependent cell death in tomato illustrate the host specificity of these pathogen effectors and raise the possibility of engineering a similar immune receptor pathway to elicit defense responses upon effector-mediated inhibition of citrus PLCPs. In addition, PLCPs themselves could be excellent targets for genetic modification. It has been shown that overexpression of a specific PLCP gene in *N*. *benthamiana* increased disease resistance to *P*. *infestans*^39^. CRISPR-based promoter editing to manipulate PLCP gene expression in citrus is another approach that could lead to urgently needed HLB resistance.

## Methods

### Plant material

Leaf and stem samples from symptomatic and asymptomatic trees were collected from mature Navel orange (*Citrus sinensis*) trees in a commercial orchard in Donna, TX and immediately frozen in liquid nitrogen. Samples were freeze-dried and sent on dry ice to the Contained Research Facility at the University of California, Davis for further processing. One-year-old Navels used for the quantitative PCR and protease inhibition assays were grown in a greenhouse at the University of California, Davis. The ambient temperature was kept at 23 °C with 72% relative humidity.

### Generation of SDE1-transgenic citrus

The 390 bp coding region of SDE1 without the signal peptide (1–24 aa) was amplified from DNA extracted from HLB-infected tissue using gene-specific primers with a start codon added to the 5′ end of the SDE1 forward primer. The PCR product was purified and cloned into pGEM-T Easy vector (Promega) and then sub-cloned into the binary vector erGFP-1380N. The recombinant vector was transformed into *Agrobacterium tumefaciens* strain EHA105 and then used for citrus transformation. Empty vector (EV) was used as a negative control.

*Agrobacterium-*mediated transformation of etiolated grapefruit epicotyl segments^45^ from the cultivar Duncan grapefruit was carried out. Epicotyls were soaked in *Agrobacterium* suspension for 1–2 min, cultured for 2 days, and then moved to a screening medium. Putative transformants were selected using kanamycin resistance and erGFP-specific fluorescence in putative transgenic lines was evaluated using a Zeiss SV11 epi-fluorescence stereomicroscope. Transgenic shoots were then micro-grafted in vitro onto 1-month-old Carrizo citrange nucellar rootstock seedlings. After 1 month of growth in tissue culture, the grafted shoots were potted into a peat-based commercial potting medium and acclimated under greenhouse conditions.

### Yeast-two-hybrid assays

A *C*. *sinensis* cDNA library was generated with total RNA extracted from healthy and *C*Las-infected tissues. The library was screened against SDE1 using a mating-based yeast-two-hybrid (Y2H) approach coupled with Illumina sequencing (performed by Qintarabio, CA). Sequences were analyzed by BLASTn using the NCBI database and top hits from *C*. *sinensis* were marked as potential SDE1-interacting proteins. Selected candidates from the Y2H screen were further tested using pairwise Y2H. The full-length cDNA of each potential SDE1interactor was cloned into the pGADT7 prey vector (Clontech) and transformed into yeast strain AH109 (Clontech) containing *SDE1* on the bait plasmid pGBKT7. Transformation of the prey plasmids into AH109 containing pGBKT7 empty vector served as a negative control.

To test the interaction of SDE1 with PLCPs of various subfamilies, cDNA sequences of the PLCP representatives *Cs*SAG12-1, *Cs*SAG12-2, *Cs*RD21a, *Cs*RD19, *Cs*AALP, *Cs*XBCP3, and *Cs*CTB, excluding their signal peptides, were cloned into pGADT7 and expressed in AH109. Signal peptides were predicted using SignalP 4.1 (organism group ‘Eukaryotes’; default D-cutoff values). For PLCP fragments encoding only the cysteine protease domain, full-length PLCP protein sequences were analyzed by SMART^46,47^ and sequences corresponding to the protease domains were cloned into pGADT7.

The experiments were repeated at least three times with similar results.

### Phylogenetic analysis of PLCPs

Protein sequences of 31 PLCP genes from *Arabidopsis thaliana*^22^ and the annotated protein sequences from the entire sequenced genome of *C*. *sinensis* were downloaded from Phytozome (https://phytozome.jgi.doe.gov/pz/portal.html). Local BLASTp with an e-value of 1e−5 was used to search for PLCP homologs in *C*. *sinensis* using the *At*PLCPs as query. To confirm that the resultant *C*. *sinensis* sequences are indeed homologous to the queried *At*PLCPs, the BLASTp search was reversed. All PLCP protein sequences were aligned using MUSCLE v3.8.3^48^. MEGA v6.06^49^ was used to construct the maximum likelihood phylogenetic tree using the James–Taylor–Thorthon model and a bootstrap value of 100.

### In vitro pull-down assays

The protease domains of *Cs*SAG12-1, *Cs*SAG12-2, *Cs*RD21a, *Cs*RD19, *Cs*AALP, *Cs*XBCP3, and *Cs*CTB were cloned into the pGEX-4T2 vector (GE Healthcare) and SDE1 was cloned into pRSF-Duet vector (gift from Dr. Jikui Song, University of California, Riverside). Vectors were transformed into *E*. *coli* BL21 cells (New England Biolabs) for protein expression. Total proteins were extracted from *E*. *coli* expressing the PLCPs and incubated with 25 µL glutathione resins (Thermo Scientific) for 1 h at 4 °C, followed by washing with TKET buffer (20 mM Tris-HCl, 200 mM KCl, 0.1 mM EDTA, 0.05% Triton X-100, pH 6.0). *SDE1*-expressing cell lysate was added to the PLCP-bound resins and incubated for 3 h at 4 °C, followed by washing to remove non-specifically bound proteins. Washed resins were boiled in Laemmli sample buffer and the supernatants were used for gel electrophoresis and the subsequent immunoblotting. The enrichment of SDE1 proteins in PLCP-bound resins was detected using an anti-SDE1 antibody^16^ followed by goat anti-rabbit-HRP (Santa Cruz). Levels of PLCPs were determined using anti-GST (Santa Cruz) followed by goat anti-rabbit-HRP (Santa Cruz). After antibody incubation, the membranes were washed, and signals were developed using SuperSignal Chemiluminescent substrates (Thermo Scientific).

For the E-64 inhibition assay, glutathione resins with bound GST-tagged *Cs*SAG12-1, *Cs*RD21a, and RCR3^34^ were incubated with either 200 μM E-64 as an inhibition treatment or TKET buffer as a control. Supernatant of *SDE1*-expressing cells was collected and incubated with the PLCP-bound resins for 3 h at 4 °C. The resins were washed and enrichment of SDE1 detected by electrophoresis and subsequent immunoblotting as described above.

The experiments were repeated at least two times with similar results. Uncropped raw data are presented in Supplementary Fig. 11.

### In vitro protease activity assay with papain

The EnzChek protease assay kit (Molecular Probes) was used to measure protease activity. Tag-free SDE1, E-64 (Sigma-Aldrich), and BSA (Gold Biotechnologies) at two different concentrations (100 and 500 nM) were mixed with papain (Sigma-Aldrich) at 100 μg/mL and added to 96-well Immulon plates (Thermo Scientific) containing BODIPY FL casein substrate. Papain with MES buffer alone served as a no treatment control for proteolytic activity and SDE2 (CLIBASIA_03230) at 300 nM served as an alternative *C*Las effector control. Reactions were allowed to perform for 1 h at room temperature in the dark before fluorescence was measured using a Tecan Pro 2000 plate reader at 460/480 nm excitation/emission, with a gain value of 50. *p*-values were determined using a two-tailed Student’s *t*-test. SDE1 and SDE2 recombinant proteins were purified from *E*. *coli* using His60 Ni-NTA Superflow resins (Clontech). The purified SDE1 proteins were cleaved with Ubiquitin-like-specific protease 1 to remove the His-SUMO tag, generating tag-free SDE1 proteins.

The experiments were repeated at least three times with similar results. Uncropped raw data are presented in Supplementary Fig. 11.

### Activity-based protein profiling

Papain (Sigma-Aldrich), *Nicotiana bethamiana* apoplastic fluids, and total citrus leaf extracts were pretreated with either buffer control, E-64, or SDE1 recombinant proteins. Total leaf extracts from *SDE1*-expressing transgenic citrus lines were pretreated with either 100 μM E-64 or buffer control. After pretreatment, the samples were incubated with a final concentration of 2 μM DCG-04^24^ for 4 h at room temperature, followed by precipitation with 100% ice-cold acetone. Samples were centrifuged at 12,000×*g*, washed with 70% acetone, then centrifuged again. Precipitated products were re-suspended in 50 mM Tris buffer (pH 6.4) and either used directly for western blotting using Streptavidin-HRP conjugates (Thermo Scientific) or further enriched on streptavidinmagnetic beads (Thermo Scientific). For enrichment, samples were incubated with 25 μL streptavidin magnetic beads at room temperature for 1 hr, washed twice with 1% SDS, and eluted by heating for 5 min at 95 °C in Laemmli sample buffer with 13% β-mercaptoethanol^50^. The labeled proteins were separated using SDS-PAGE and active proteases were visualized by western blotting using Streptavidin-HRP conjugates (Thermo Scientific).

The experiments were repeated two times with similar results. Uncropped raw data are presented in Supplementary Fig. 11.

### Gene expression analyses using qRT-PCR

One-year-old *C*. *sinensis* (Navel) trees grown in the greenhouse were sprayed with 2 mM salicylic acid (SA) or water with 0.02% of Silwet L-77 as an adjuvant. After 48 h, fully expanded young leaves were harvested, flash frozen in liquid nitrogen, and stored at −80 °C. A total of three trees (biological replicates) were analyzed for each treatment.

Total RNA was extracted using a Trizol (Invitrogen)-based method. 1.5 mg RNA in a 20 μL reaction was used for cDNA synthesis using M-MLV reverse transcriptase (Promega). The CFX96 real-time PCR detection system (Bio-Rad) was used to assess PLCP gene expression. Quantitative reverse transcription PCR reactions used Bio-Rad SoFast EvaGreen Supermix according to the manufacturer’s directions. Thermocyling began with a first step at 95 °C for 30 s followed by 40 cycles alternating between 5 s at 95 °C and 15 s at 60 °C. A melting curve was performed after the final cycle and ran 5 s at 65 °C and 5 s at 95 °C. Gene expression was normalized to the Cyclooxygenase (*COX*, KF933043.1) gene^51^. All primers, gene names, and accession numbers are provided in Supplementary Data 1.

This experiment was repeated three times with similar results.

### Citrus imprint assay

Freshly cut stems of *C*Las-infected (both symptomatic and asymptomatic) Rio Red grapefruit trees from a commercial orchard in Donna, TX and healthy (*C*Las-free) stems from grapefruit kept in a screen house were imprinted onto nitrocellulose membranes. *C*Las status was verified by qRT-PCR prior to imprinting. Imprinted membranes were then incubated with either anti-AALP (gift from Dr. Natasha Raikhel, University of California, Riverside) or anti-SDE1 antibodies^16^ and the corresponding proteins were detected using goat anti-rabbit-HRP secondary antibodies (Santa Cruz) and SuperSignal Chemiluminescent substrates (Thermo Scientific).

The experiments were repeated two times with similar results.

### Mass spectrometry analyses of PLCP abundance and activity

To assess for PLCP abundance, a total of 250 μg of uninfected and infected leaf extract was ground in 50 mM Tris (pH 6.8) and 2 µM DTT in a total reaction volume of 500 µL. Protein extracts were divided for the detection of activity (below) and PLCP abundance. Proteins were precipitated as described above for the ABPP assay. The protein pellet was re-suspended in 8 M urea in 100 mM ammonium bicarbonate (ABC). The samples were reduced and alkylated with 10 mM DTT and 30 mM of iodoacetamide (IAA) in 100 mM ABC for 1 h, respectively. Samples were then diluted to a final concentration of 1 M urea by adding 100 mM ABC. Two micrograms of trypsin were added and the samples incubated overnight at 37 °C. The tryptic digest was arrested by lowering the pH to ≤3 with formic acid. Peptide desalting and purification was performed with the MacroSpin C18 column protocol (The Nest Group).

To determine PLCP activity, the other half of the leaf extracts from above were incubated with a final concentration of 2 μM DCG-04 for 4 h at room temperature and precipitated as describes above for the ABPP assay, followed by further enrichment of the DCG-04 labeled products on streptavidin beads. Beads were washed three times with 50 mM ABC. Samples were reduced with 50 mM DTT for 1 h at 60 °C and alkylated with 50 mM IAA for 1 h at room temperature. Tryptic on-bead digests were performed with 250 ng of trypsin and the samples incubated at 37 °C overnight. The digests were arrested by adding 50 µL 60% acetonitrile/0.1% trifluoroacetic acid to the resins and incubating for 10 min at room temperature.

Peptides were submitted to the Proteomics Core of the Genome Center at the University of California, Davis for liquid chromatography-MS/MS. The LC-MS/MS system configuration consisted of a CTC Pal autosampler (LEAP Technologies) and Paradigm HPLC device (Michrom BioResources) coupled to a QExactive hybrid quadrupole Orbitrap mass spectrometer (Thermo Scientific) with a CaptiveSpray ionization source (Michrom BioResources). Peptides were analyzed by as described below^52^. Peptides were reconstituted in 2% acetonitrile and 0.1% formic acid and were washed on a Michrom C18 trap then were eluted and separated on a Michrom Magic C18AQ (200 µm × 150 mm) capillary reverse-phase column at a flow rate of 3 µL/min. A 120 min gradient was applied with a 2% to 35% B (100% acetonitrile) for 100 min, a 35% B to 80% B for 7 min and 80% B for 2 min. Then a decrease of 80% to 5% B in 1 min followed by 98% A (0.1% formic acid) for 10 min. The QExactive was operated in Data-Dependent Acquisition (DDA) mode with a top-15 method. Spray voltage was set to 2.2 kV. The scan range was set to 350–1600 *m*/*z*, the maximum injection time was 30 ms and automatic gain control was set to 1 × 10^6^. Precursor resolution was set to 70,000. For MS/MS, the maximum injection time was 50 ms, the isolation window was 1.6 *m*/*z*, the scan range 200–2000 *m*/*z*, automatic gain control was set to 5 × 10^4^ and normalized collision energy was 27%. The dynamic exclusion window was set to 15 s and fragment product resolution was 17,500. An intensity threshold of 1 × 10^4^ was applied and the underfill ratio was 1%.

Peptide identification, analyses, and quantification: The raw data files were imported into MaxQuant v1.5.6.5^53^ for label-free intensity-based quantification. The database search engine Andromeda^54^ was used to search MS/MS spectra against the *C*. *clementina* and *C*. *sinensis* databases downloaded from Phytozome with a tolerance level of 20 ppm for the first search and 6 ppm for the main search. Trypsin/P was set as the enzyme and two missed cleavages were allowed. Protein N-terminal acetylation, Methionine oxidation, and NQ deamidation were set as variable modifications. The maximum number of modifications per peptide was set as five and contaminants were included. The ‘match between runs’ feature was checked with a match time window of 0.7 min and an alignment time window of 20 min. The FDR for protein level and peptide spectrum match (PSM) was set to 1%. The minimum peptide length was 6, minimum razor and unique peptides was changed to 0, and minimum unique peptides was set to 1. The minimum ratio count for protein quantification was set to 2.

To ensure that abundance and activity data were analyzed separately, the “Separate LFQ in parameter groups” option in the global parameters tab was selected. This option allows MaxQuant to perform retention time alignments and calculate a normalization factor for abundance and activity separately. The other MaxQuant settings were left as default. The total peptide intensities for each replicate were summed and a normalization factor was calculated for each sample^55^. This normalization factor was applied based on the least overall proteome change. Peptide ratios between samples were then calculated to obtain a pair-wise protein ratio matrix between samples, which was subsequently used to rescale the cumulative intensity in each sample and provides the label-free intensity (LFQ) value^55^. A description of identified peptides in all analyses is included in Supplementary Data 2 and raw MS data have been deposited in PRIDE (http://www.ebi.ac.uk/pride/archive/projects/PXD008366)^56^. The MaxQuant output file was imported into Perseus 1.5.015^57^. Potential contaminants, reverse hits, and proteins identified only by modified peptides were excluded. The LFQ intensities were log_2_-transformed. Proteins not consistently identified in at least two out of the three replicates in at least one group were discarded. Missing values were substituted with values from a normal distribution of the obtained intensities using default settings (width 0.5, downshift 1.8). Differentially changing proteins were identified using a two-tailed Student’s *t*-test. A *p*-value of less than 0.05 was used for truncation.

### Structural model of *Cs*SAG12-1

The protein sequence for the catalytic domain of *Cs*SAG12-1 was submitted to ModWeb^58^ (https://modbase.compbio.ucsf.edu/modweb/) using the default settings. The template used for *Cs*SAG12-1 was CysEP from *Ricinus communis* (RCSB Protein Data Bank ID 1S4V) with 56% sequence identity. Molecular graphics images were produced using the UCSF Chimera package from the Resource for Biocomputing, Visualization, and Informatics at the University of California, San Francisco (supported by NIH P41 RR-01081). The Chimera interactive graphics modeling program was used to view and compare structures^59^.

### *Pseudomonas syringae* infection assay

The leaves of 5-week-old *Arabidopsis thaliana* plants (ecotype Col-0) were infiltrated with bacterial suspensions at OD_600_ = 0.0001 (~1 × 10^5^ cfu/mL). The inoculated plants were transferred to a growth chamber (22 °C, 16/8 h light/dark regime, 90% relative humidity), and the bacterial populations were determined as colony forming units (cfu) per cm^2^ 3 days after inoculation^33^. To induce *SDE1* expression under the *hopZ1a* promoter, *P*. *syringae* strains were grown in M63 minimal medium containing 1% fructose^60^ at room temperature for 24 h. The bacterial cells were then collected by centrifugation and re-suspended in 10 mM MgSO_4_ buffer for inoculation using needle-less syringes.

The experiments were repeated three times with similar results.

### Cf-2-mediated cell death in tomato

Full-length *Avr2* was synthesized using gBlocks Gene Fragments (Integrated DNA Technologies). Primers were designed to add a 6xHIS tag at the N-terminus of the mature protein (no signal peptide) and the resultant fragment cloned into pFLAG-ATS (Sigma-Aldrich) (F: 5′-GTA AAG CTT CAC CAT CAC CAT CAC CAT GCC AAG AAA TTA-3′, R: 5′-CTG AGA TCT CAA CCA CAA AGT CC-3′). The construct was transformed into *E*. *coli* BL21 for protein expression. Recombinant proteins were purified using Ni-NTA agarose (Qiagen) and dialyzed into water. SDE1 was purified as described above. Three different concentrations (10 nM, 100 nM, and 2 µM) of purified Avr2 and SDE1 recombinant proteins were syringe infiltrated into 3-week-old leaves of tomato cultivar Moneymaker. The following near-isogenic lines were used:^25^ *Cf-2/RCR3*^*pim*^, *Cf-2/rcr3-3*, and Cf-0. Images were taken 7 days after infiltration.

The experiments were repeated two times with similar results.

### Statistical data analysis

The biological data reported in this study was analyzed as follows using SAS JMP Pro v13.0. To test for normal distribution of the collected data, normal quantile plots were inspected and Shapiro–Wilk goodness-of-fit tests were performed. To ensure that the variances are equal, the Levene’s test was used. When comparing a test group to a control group, a two-sided Student’s *t*-test was used. The significance values are reported as follows: * = *p* *<* 0.05, ** = *p* *<* 0.01, and *** = *p* *<* 0.001. When comparing the means of multiple groups, a one-way ANOVA followed by Tukey’s HSD post hoc test was performed. Significant differences between groups (*p* < 0.05) are denoted with different letters.

### Antibodies and chemicals

Streptavidin-HRP used for ABPP of PLCPs was purchased from ThermoFisher (Cat. No. 21130) and used in 1:1000 dilution. Antibodies used in this study include Goat-anti-Rabbit IgG-HRP (Santa Cruz, Cat No. SC2004, used in 1:5000 dilution), Anti-AALP (anti-serum gifted from Dr. Natasha Raikel in ref. ^30^, used in 1:1000 dilution), Anti-SDE1 (polyclonal antibody generated in ref. ^16^, used in 1:1500 dilution), Anti-GST (Santa Cruz, Cat No. SC138, used in 1:2000 dilution), Anti-HA high affinity (Roche, Cat No. 11867423001, used in 1:1500 dilution), Goat-anti-Rat IgG-HRP (Santa Cruz, Cat No. SC2065, used in 1:5000 dilution).

### Data availability

The mass spectrometry data generated in this study has been deposited in the PRIDE Archive under accession number PXD008366. The authors declare that all other data supporting the findings of this study are available within the manuscript and its supplementary files or are available from the corresponding authors upon request.

## Electronic supplementary material

Supplementary Information

Description of Additional Supplementary Files

Supplementary Data 1

Supplementary Data 2
