# Supplementary material for: An effector from the Huanglongbing-associated pathogen targets citrus proteases
Source: Nat Commun. 2018 Apr 30;9:1718. doi: 10.1038/s41467-018-04140-9 (PMC5928222; doi:10.1038/s41467-018-04140-9)
Supplement: Supplementary file 1 — Supplementary Information [file 41467_2018_4140_MOESM1_ESM.pdf]

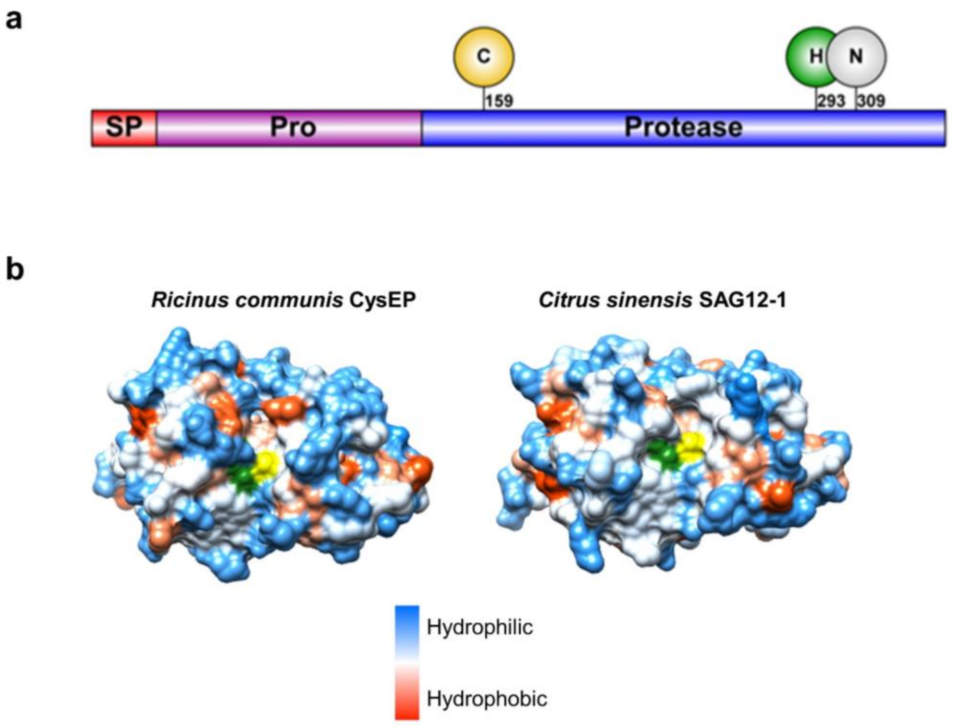

Supplementary Figure 1. PLCP domain architecture and structural model of SAG12-1 from *Citrus sinensis*. a, Canonical PLCPs possess a signal peptide (SP), an autoinhibitory pro-peptide, and the catalytic protease domain. The predicted catalytic triad of papain (cysteine159, histidine293 and asparagine309) are presented as an example. **b,** A structural model for *Cs*SAG12-1. The predicted protease domain of *Cs*SAG12-1 was submitted to MODELLER, revealing 56% sequence similarity to the CysEP PLCP from *Ricinus communis* (PDB: 1S4V). PDB 1S4V was used as a template for structural modeling of *Cs*SAG12-1 and visualized in CHIMERA.

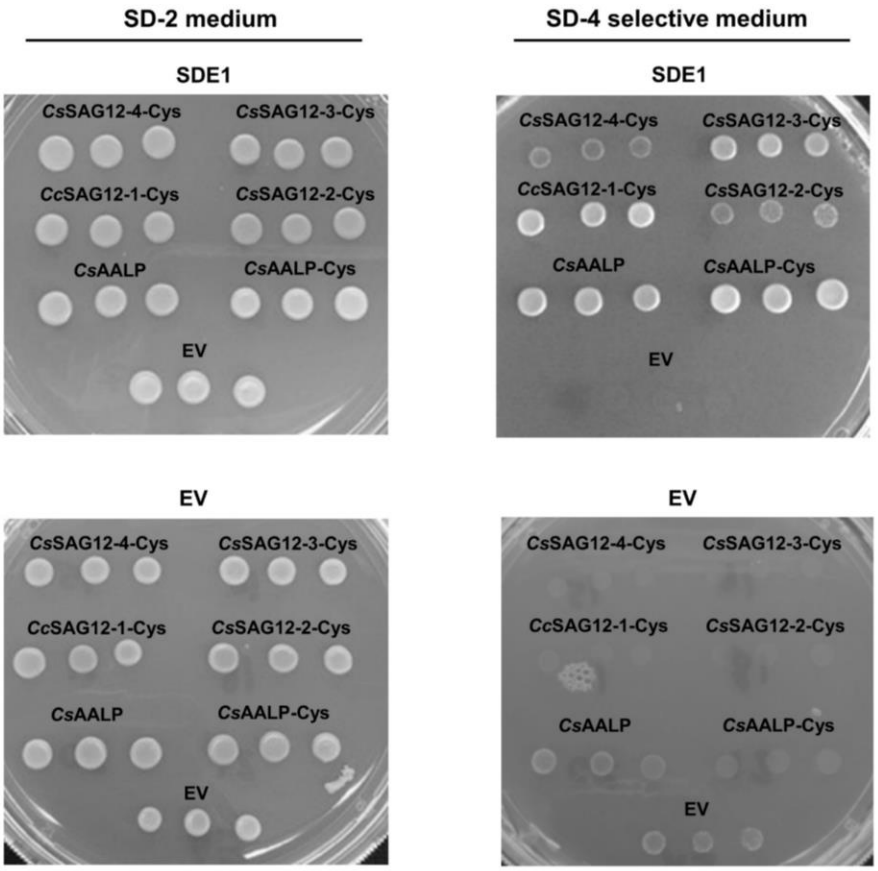

Supplementary Figure 2. SDE1 interacts with additional PLCPs from the SAG12 subfamily. Pairwise yeast-two-hybrid (Y2H) assay using SDE1 as bait and the cysteine protease domains of PLCPs (*Cs*SAG12-1, *Cs*SAG12-3, *Cc*SAG12-4) as the prey. Growth of yeast on SD-4 selective medium represents protein-protein interaction, growth of yeast on SD-2 medium confirms yeast transformation. Yeast cells transformed with pGBKT7 and pGADT7 empty vectors severed as negative controls. *Cs*AALP (full-length) and *Cs*AALP-Cys (cysteine protease domain only) served as positive controls.

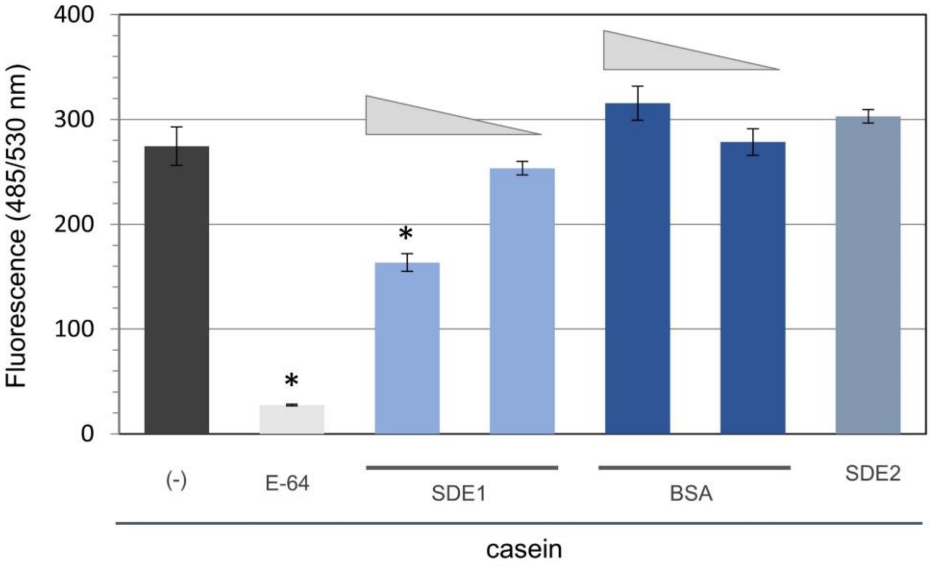

Supplementary Figure 3. SDE1 but not SDE2 can inhibit the protease activity of papain. Proteolytic activity of papain measured by digestion of fluorescent casein substrates in the presence of 1 μM E-64, purified SDE1 (0.74 and 0.15 μM) or SDE2 (0.3 μM) protein (CLIBASIA_03230), and BSA (0.74 and 0.15 μM). Fluorescence was measured at 485 nm excitation over 530 nm emission. Values are average of duplicates with the Standard Deviation shown as the error bars. Statistical analysis was done using Student's two-tailed t-test and significant differences ($p < 0.05$) are labeled with asterisks (*).

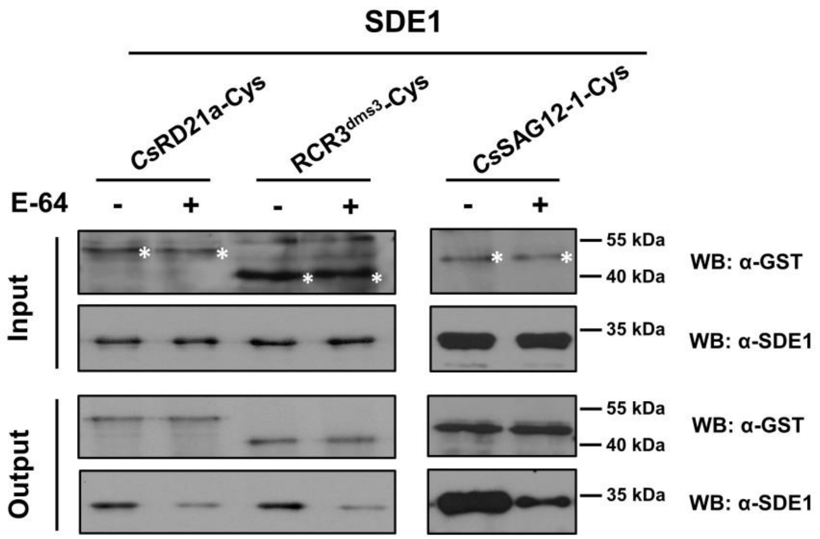

Supplementary Figure 4. **E-64 inhibits the interaction of SDE1 with PLCPs *in vitro*.** Proteins extracted from *E. coli*-expressing GST-tagged cysteine protease domains of *Cs*RD21a, *Cs*SAG12-1 or RCR3^{dms3} (from the wild potato species *Solanum demissum*) were pre-incubated with E-64. The enrichment of SDE1 on PLCP-bound resins was then examined by western blotting using an anti-SDE1 antibody. Asterisks (*) indicate the protein bands corresponding to individual PLCPs.

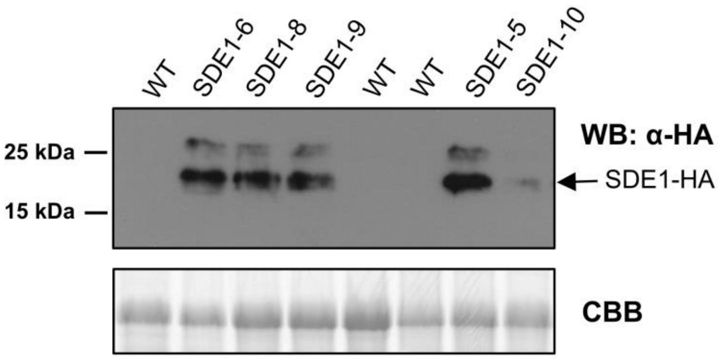

Supplementary Figure 5. SDE1 proteins accumulate in transgenic citrus seedlings. Leaves from individual transgenic citrus lines (one-year-old seedlings) were ground with liquid nitrogen into powder and re-suspended in 2x Laemmli loading dye. Samples were boiled for five minutes, then separated on a 12% protein gel for western blotting. SDE1 proteins were detected by anti-HA antibody (Santa Cruz Biotechnology, CA). Gel stained with Coomassie brilliant blue (CBB) served as a loading control. Leaf tissue from wild-type (WT) grapefruit seedlings of the same age were included as controls.

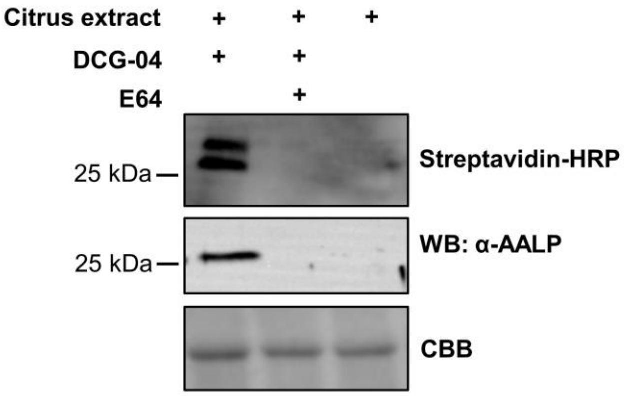

Supplementary Figure 6. The anti-AALP antibody specifically recognizes citrus PLCPs. Two-hundred fifty micrograms of citrus leaf extract were incubated with and without E-64 for 30 min prior to the addition of DCG-04 for ABPP. Active PLCPs were captured by streptavidin IP coupled with western blotting using streptavidin-HRP or anti-AALP antibodies. Coomassie brilliant blue (CBB) stained gel served as a loading control.

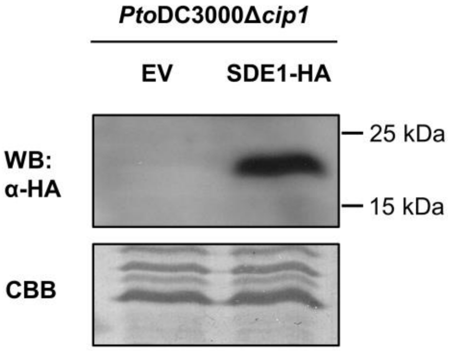

Supplementary Figure 7. SDE1 is expressed and secreted by *Pseudomonas syringae* in an inducible medium. *SDE1-HA* (full-length gene including sequences corresponding to the N-terminal secretion signal) was cloned in the plasmid vector pUCP20tk containing the promoter of *hopZ1a*. Empty vector and pUCP20tk::*SDE1* were introduced into *P. syringae* pv. tomato DC3000 Δ*cip1* knockout mutant by electroporation. Transformants were grown in the M63 minimal medium (pH 5.3) to induce *SDE1* expression. The secretion of SDE1 proteins was detected in the supernatant of the cell culture using western blotting. Coomassie brilliant blue (CBB) stained gel served as a loading control.

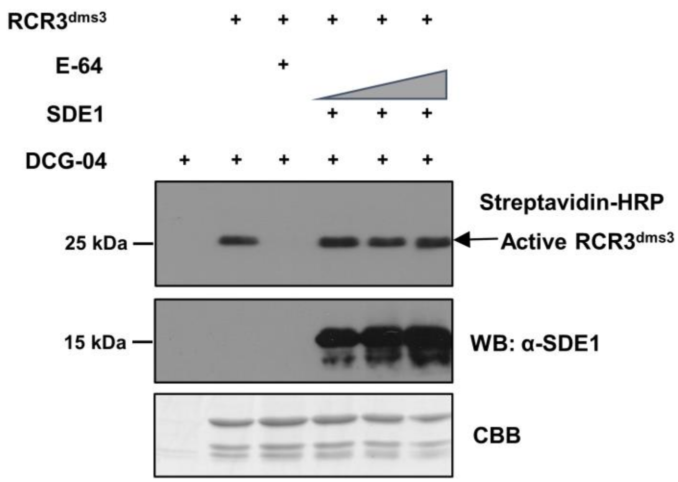

Supplementary Figure 8. SDE1 does not inhibit the activity of the wild potato RCR3^{dms3}. Full-length of RCR3 from the wild potato species *Solanum demissum* (RCR3^{dms3}) was transiently expressed in *N. benthamiana* and secreted into the apoplast. Apoplastic fluid was extracted and the active proteases were labeled using DCG-04 in the presence of 0.8, 1.6 or 3.2 μM of purified SDE1 protein. Coomassie brilliant blue (CBB) served as a loading control.

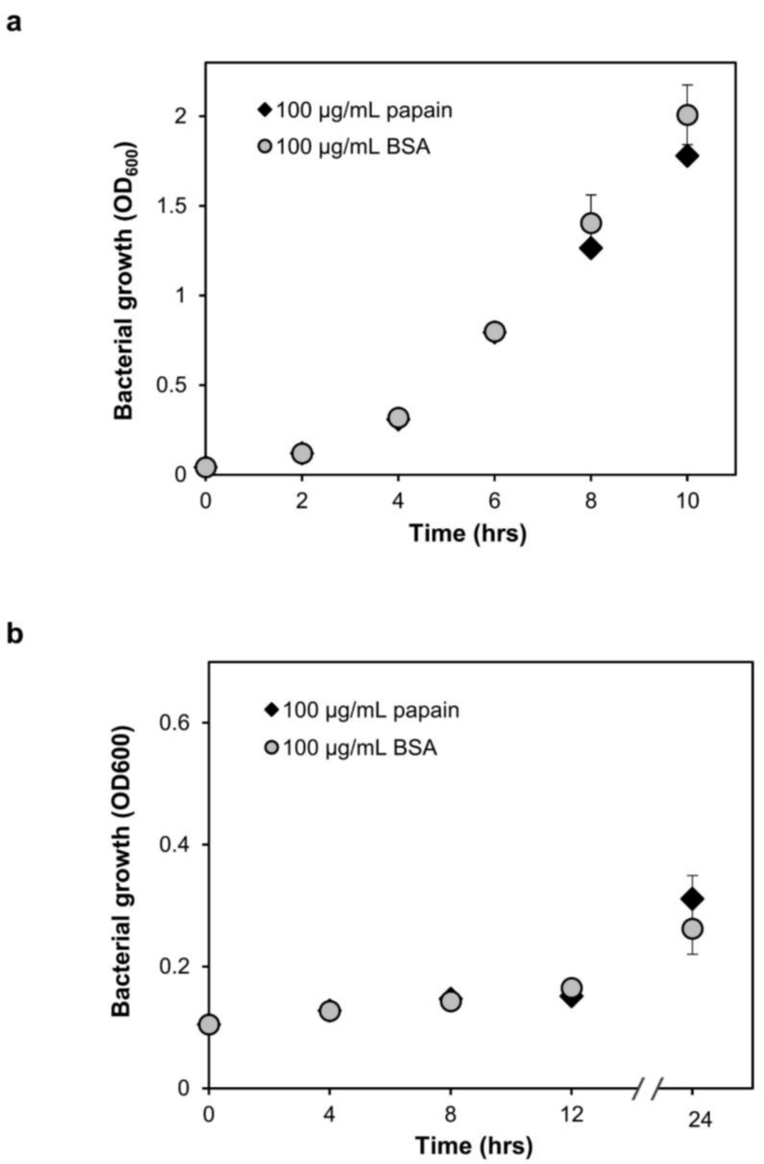

Supplementary Figure 9. Supplementation of papain in culture media does not inhibit the growth of *Pto*DC3000Δ*cip1*. *Pto*DC3000Δ*cip1* containing pUCP20tk empty vector was grown in **a,** King's B medium or **b,** hrp-inducing minimal medium in the presence of either 100 µg/mL papain (black diamonds) or 100 µg/mL BSA (grey circles) and the optical density of the cultures (OD_{600}) was monitored over time. Graphs show mean ± standard deviation of three replicates. This experiment was performed twice with similar results.

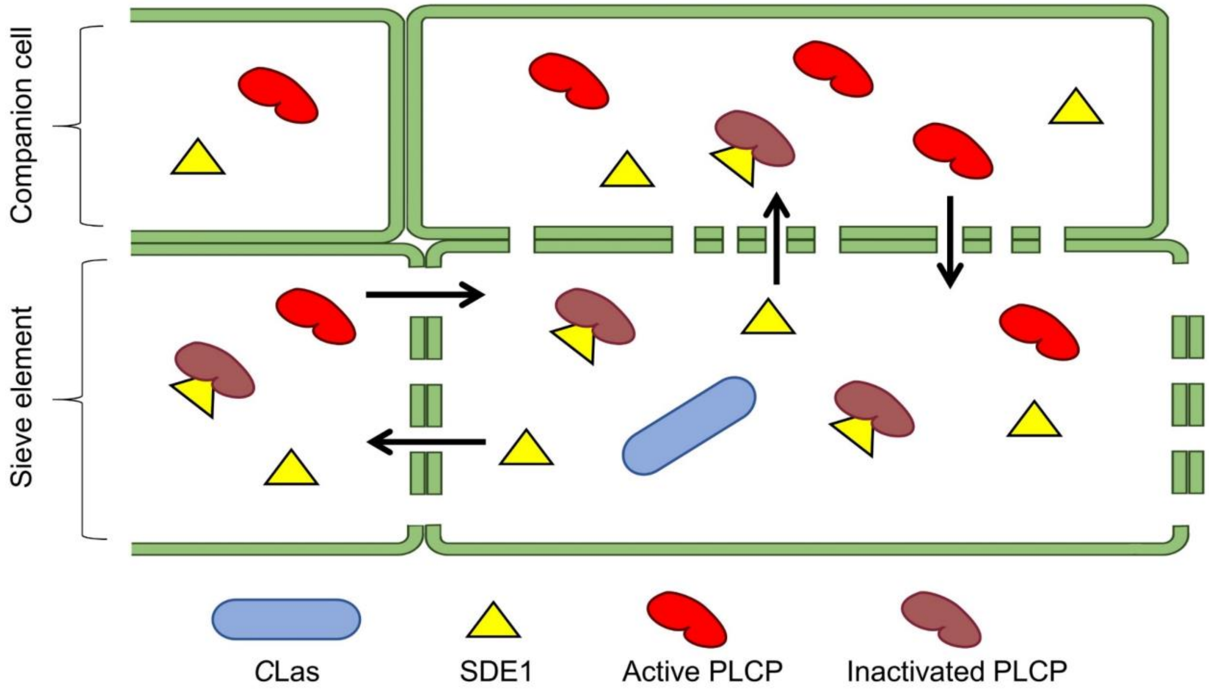

Supplementary Figure 10. A potential model of SDE1 and PLCP interaction in *C*Las-infected citrus. After infection, *C*Las proliferates in phloem sieve elements. Sieve elements are dependent upon adjacent, metabolically active companion cells. Citrus is able to perceive the bacterial pathogen and induce defense responses, including increased PLCP accumulation. These proteins may be directly delivered into the sieve elements through plasmodesmatal connections. *C*Las possesses the Sec secretion system and secretes multiple Sec-delivered effectors, including SDE1, which acts to inhibit the protease activity of PLCPs. SDE1 can move through the sieve elements and may be able to translocate into adjacent companion cells to suppress this PLCP-based defense responses and promote bacterial infection.

Figure 1d

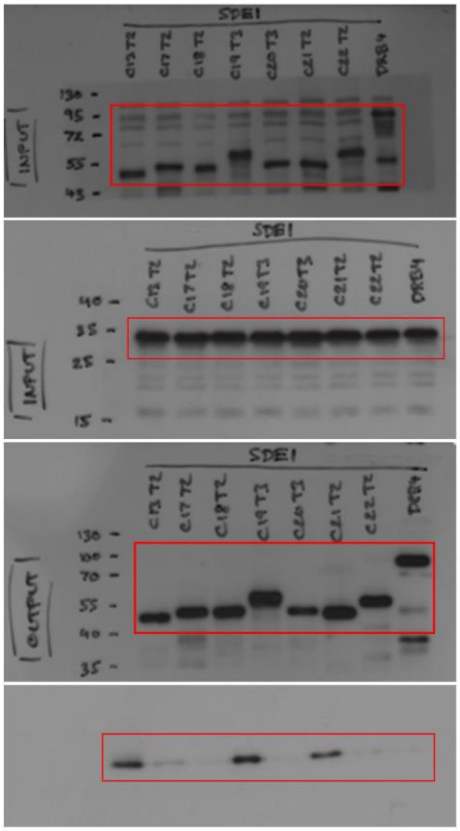

Figure 2b

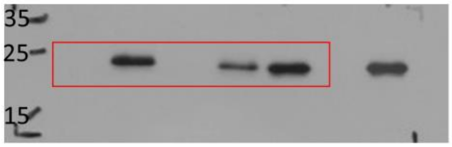

Figure 2c

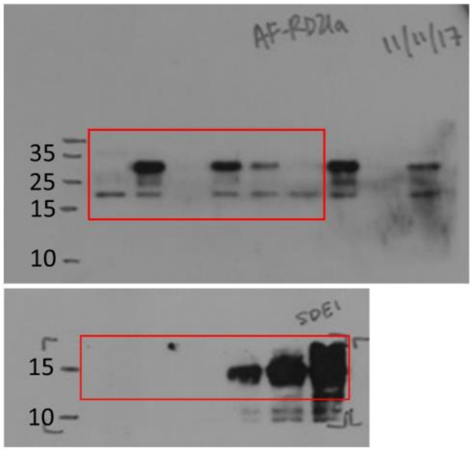

Figure 2d

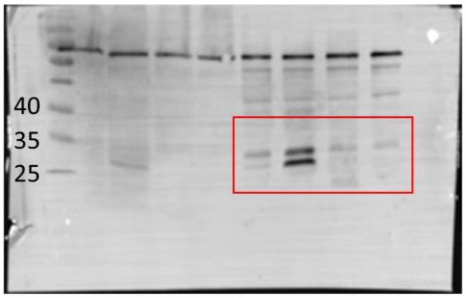

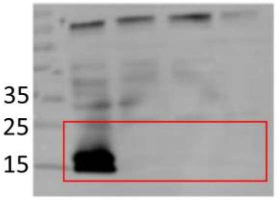

These two blots were flipped horizontally for presentation in order to be consistent with the sample loading orders in other experiments.

Figure 2e

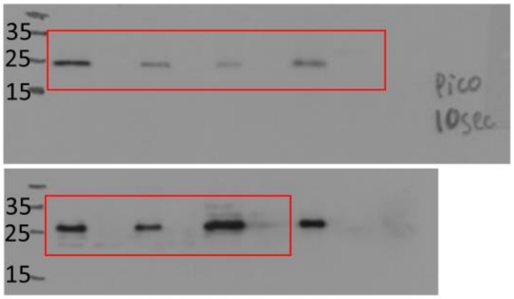

Figure 6b

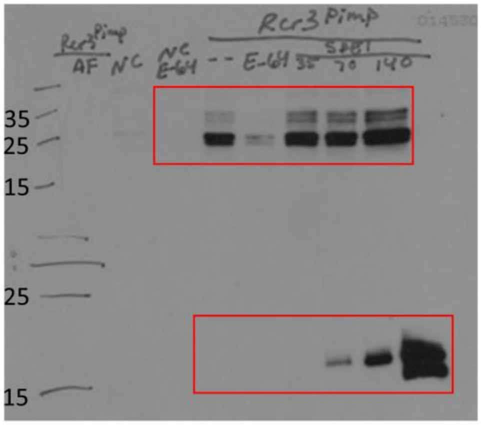

Supplementary Figure 11. Un-cropped raw western blotting data presented in the main figures.

Supplementary Table 1. Candidates identified from yeast-two-hybrid screening using SDE1 as the bait. Only “xylem cysteine proteinase 1” was confirmed using pairwise Y2H assay.

SDE1-interacting candidate	NCBI Sequence ID	Pairwise Y2H Positive
Diacylglycerol (DAG) protein, chloroplastic	XM_006475194	No
E3 ubiquitin−protein ligase RNF12−B	XM_006489385	No
Putative E3 ubiquitin−protein ligase XBAT31	XM_006488247	No
Heavy−metal−associated domain−containing protein	XM_006467144	No
Calcyclin−binding protein	XM_006467400	No
Xylem cysteine proteinase 1	XM_006493578	Yes

Supplementary Table 2. Analysis of published transcriptome data showing differentially expressed *PLCP*s in *C*Las-infected *C. sinensis.*

Gene ID in *C. sinensis*	PLCP subfamily classification	Log_2FC[1]	FDR[2]	Source
orange1.1g018568m	CTB	-1.93764	N/A	Martinelli et. al. (2012) RNA-seq
orange1.1g047264m	XCP1	-1.07107	N/A	Martinelli et. al. (2012) RNA-seq
orange1.1g017419m	RD21a	1.1742	N/A	Martinelli et. al. (2012) RNA-seq
orange1.1g012960m	XBCP3	1.43348	N/A	Martinelli et. al. (2012) RNA-seq
orange1.1g036910m	AALP	1.54139	N/A	Martinelli et. al. (2012) RNA-seq
orange1.1g024783m	RD21a	1.59572	N/A	Martinelli et. al. (2012) RNA-seq
orange1.1g019063m	SAG12	1.97224	N/A	Martinelli et. al. (2012) RNA-seq
orange1.1g019112m	SAG12	2.6825	N/A	Martinelli et. al. (2012) RNA-seq
orange1.1g018781m	XCP1	0.822224	N/A	Martinelli et. al. (2012) RNA-seq
orange1.1g017318m	RD19	0.916496	N/A	Martinelli et. al. (2012) RNA-seq
orange1.1g012960m	XCBP3	0.812	3.08E-15	Kim, J.-S. et. al. (2009) Microarray
orange1.1g017419m	RD21a	0.815	0.0438	Kim, J.-S. et. al. (2009) Microarray
orange1.1g018104m	CEP1	1.384	1.82E-06	Kim, J.-S. et. al. (2009) Microarray
orange1.1g018958m orange1.1g018968m	SAG12	3.192	3.08E-15	Kim, J.-S. et. al. (2009) Microarray

[1] Log_2 fold-change

[2] False discovery rate

Martinelli, F. *et al.* Transcriptome Profiling of Citrus Fruit Response to Huanglongbing Disease. *PLos One* **7**, e38039 (2012).

Kim, J.-S. *et al*. Response of sweet orange (*Citrus sinensis*) to '*Candidatus* Liberibacter asiaticus' infection: microscopy and microarray analyses. *Phytopathology* **99**, 50-57 (2009).
