# Supplementary material for: An effector from the Huanglongbing-associated pathogen targets citrus proteases
Source: Nat Commun. 2018 Apr 30;9:1718. doi: 10.1038/s41467-018-04140-9 (PMC5928222; doi:10.1038/s41467-018-04140-9)
Supplement: Supplementary file 2 — Description of Additional Supplementary Files [file 41467_2018_4140_MOESM2_ESM.pdf]

Descriptions of Additional Supplementary Files

Supplementary Data 1: Primers used in this study.

Supplementary Data 2: PLCP peptides identified by Mass Spectrometry in Figure 4.
